# Can endopolyploidy explain body size variation within and between castes in ants?

**DOI:** 10.1002/ece3.623

**Published:** 2013-06-04

**Authors:** Daniel R Scholes, Andrew V Suarez, Ken N Paige

**Affiliations:** 1 Program in Ecology Evolution and Conservation Biology, University of Illinois at Urbana-Champaign515 Morrill Hall, 505 S Goodwin Ave, Urbana, Illinois, 61801; 2 Department of Entomology, University of Illinois at Urbana-Champaign515 Morrill Hall, 505 S Goodwin Ave, Urbana, Illinois, 61801; 3 Department of Animal Biology, University of Illinois at Urbana-Champaign515 Morrill Hall, 505 S Goodwin Ave, Urbana, Illinois, 61801

**Keywords:** Ants, body size, caste, endoreduplication, ploidy

## Abstract

Endoreduplication is the process by which the nuclear genome is repeatedly replicated without mitotic cell division, resulting in nuclei that contain numerous additional genome copies. Endoreduplication occurs widely throughout Eucarya and is particularly common in angiosperms and insects. Although endoreduplication is an important process in the terminal differentiation of some specialized cell types, and often increases cell size and metabolism, the direct effects of increasing nuclear ploidy on cell function are not well resolved. Here, we examine if endoreduplication may play a role in body size and/or caste differentiation in ants. Nuclear ploidy was measured by flow cytometry of whole individuals (providing the basis for overall body size patterns) and individual body segments for multiple polymorphic ant species. We used cell cycle values, interpreted as the mean number of endocycles performed by each cell in the sample, as our measure of overall endoreduplication. Among females of four polymorphic ant species, endoreduplication was positively related with size within the worker caste, but was not related to caste generally in two species where we also examined queens. Additionally, abdomens had the greatest endoreduplication of all body parts regardless of caste or size. We also found that males, having derived from haploid unfertilized eggs, had the highest rates of endoreduplication and may compensate for their haploid origin by performing an additional endocycle relative to females. These results suggest that endoreduplication may play a role in body size variation in eusocial insects and the development of some segment-specific tissues.

## Introduction

Endoreduplication is the process by which the nuclear genome is repeatedly replicated without mitotic cell division, resulting in a mosaic of nuclear ploidy levels within the organism (termed endopolyploidy). High levels of nuclear ploidy are most commonly observed in fully differentiated and highly specialized cell types throughout Eucarya (Nagl 1976), and it is hypothesized that changes in cell size, structure, internal composition, and potentially gene regulation result from the process of endoreduplication (Bennett 1972; Nagl 1976). These changes, collectively termed “nucleotypic effects” (Bennett 1972), may be inherently beneficial to a cell's differentiation or specialization through improving the cell's rate of water and nutrient uptake, transport efficiency, and storage capacity (Barow 2006; Lee et al. 2009).

While endoreduplication is most common in specialized cell types, endopolyploidy is also common throughout the bodies of individuals of many angiosperms and selected animal families (Brodsky and Uryvaeva 1977; Barow 2006). For these individuals, endoreduplication may be particularly important in overall organismal growth, providing an energetically efficient means of increasing organism or tissue size through cell growth without requiring mitosis and the accompanying cytoskeletal rearrangement of cell division (Kondorosi et al. 2000). For example, in *Arabidopsis thaliana* increased endopolyploidy is related to increases in biomass and seed production following herbivory, which shortens the annual plant's growing season and necessitates rapid and efficient regrowth (Scholes and Paige 2011). Endoreduplication has also been related to overall body size in the nematode *Caenorhabditis elegans* (Flemming et al. 2000; Lozano et al. 2006), where the experimental increase or decrease of endopolyploidy results in an increase or decrease, respectively, in adult body size. These examples together provide correlative evidence of endopolyploidy's role in influencing overall body size generally, although more substantive evidence of a direct relationship remains elusive, particularly in animal systems where systemic endopolyploidy is not well documented.

Here, we examine if endopolyploidy is related to variation in caste and body size in ants. The eusocial Hymenoptera are often characterized by extreme variation in body size, particularly between reproductive and worker castes (Hölldobler and Wilson 1990). Variation in the size, number, and behavior of individuals within and among castes provides the basis for the division of labor that is a hallmark of the success of social insects (Wilson 1974; Oster and Wilson 1978; Robinson 1992), where specialization of individuals is analogous with cell specialization such that the colony functions as a “superorganism” (Hölldobler and Wilson 2009). Reproductive and nonreproductive castes vary considerably in terms of tissue-specific growth. Notably, reproductives (both males and new queens) usually have highly developed wing muscles, reproductive organs, and a battery of glands that are often atrophied or missing in workers (Chapman 1998). In addition to these caste-specific differences, in many ants workers are also highly polymorphic and can be subdivided into subcastes that vary both in overall size and the degree of morphological specialization (Wilson 1953; Oster and Wilson 1978). Much like reproductive individuals, specialized workers may have specific body parts and organs that are disproportionately large. For example, the largest workers in some species have disproportionately large heads and may act as soldiers, seed millers, or “living plugs” in which their highly modified heads are used to block the entrance to the colony (Hölldobler and Wilson 1990; Stapley 1999; Powell 2008).

For most ants, morphological variation between and within castes results from environmental cues, specifically the amount and type of nutrition received during larval development (Wheeler 1986, 1991; but see Schwander et al. 2010 for exceptions). These cues then influence developmental pathways related to nutrition, growth, metabolism, and reproduction (Smith et al. 2008; Rajakumar et al. 2012). Highly specialized tissues that develop in queens (e.g., flight muscles, reproductive organs, chemically rich glands) and the largest workers (e.g., enlarged mandibular muscles) may in turn have high metabolic demands. Although never tested, the energetic requirements of these tissues may be met, in part, by the genetic and/or nucleotypic effects of endoreduplication (Nagl 1978; D'Amato 1989; Aron et al. 2005).

Similarly, mechanisms regulating cellular metabolic output may also be particularly important in the context of the haplodiploid sex determination system of the order Hymenoptera. In this system, fertilized eggs laid by mated queens develop into diploid females, whereas unfertilized eggs become haploid males, although diploid males can occur in colonies with low genetic diversity (Cook and Crozier 1995; Packer and Owen 2001). The haploidy of males' cells could have severe consequences on the absolute metabolism of specialized tissues (Epstein 1964, 1967) such as those related to their reproductive success, including dispersal capability (via the metabolism of the flight muscle cells) and sperm production (via the metabolism of the cells that support gametogenesis). Haploidy could therefore be a sex-specific handicap unless a mechanism such as endoreduplication could increase cell size, organellar metabolic potential, and gene copy number in proportion to their female counterparts (Aron et al. 2005).

In this study, we examine endopolyploidy in relation to variation in body size among workers of four species of ant. We then compare worker endopolyploidy to that of queens in two species to test for differences in endopolyploidy between reproductive and nonreproductive females. For one of these species (*Solenopsis invicta*), we also measure ploidy levels in males to determine if their haploid origin results in any sex-specific patterns in endopolyploidy. We additionally examine variation in endopolyploidy levels among the three major body parts (head, thorax, and abdomen) in a preliminary effort to determine which tissues may be driving differences in caste, sex, and/or body size seen. Overall, our approach aims to provide preliminary insight into the potential for endoreduplication to contribute to body size differences within and among castes in ants, possibly by its associated metabolic and nucleotypic effects.

## Methods

### Study species and comparisons

To examine the role of endoreduplication on ant body size and caste, we used whole individuals of four species of ant that exhibit considerable variation in worker body size: *S. invicta* (red imported fire ant), *Pogonomyrmex badius* (Florida harvester ant), *Camponotus floridanus* (Florida carpenter ant), and *Atta texana* (Texas leaf-cutting ant) (Table 1). The *S. invicta* colonies collected were polygynous, while colonies of *P. badius*, *C. floridanus*, and *A. texana* were monogynous. Numerous alate queens were present in the *S*. *invicta* and *P. badius* colonies. While the largest workers of each of these species can have a distinct morphology (often called “majors” and considered their own subcaste), all of these species show nearly continuous variation in body size between their smallest and largest workers with the exception of *P. badius* (which is often reported as being dimorphic, with distinct “major” and “minor” workers, rather than continuously polymorphic). In this study, for consistency, we use the term “type” where relevant to discuss the various groups sampled within an ant colony (males: M, queens: Q, large workers: LW, small workers: SW).

**Table 1:** Samples analyzed for nuclear DNA content by flow cytometry for each species studied

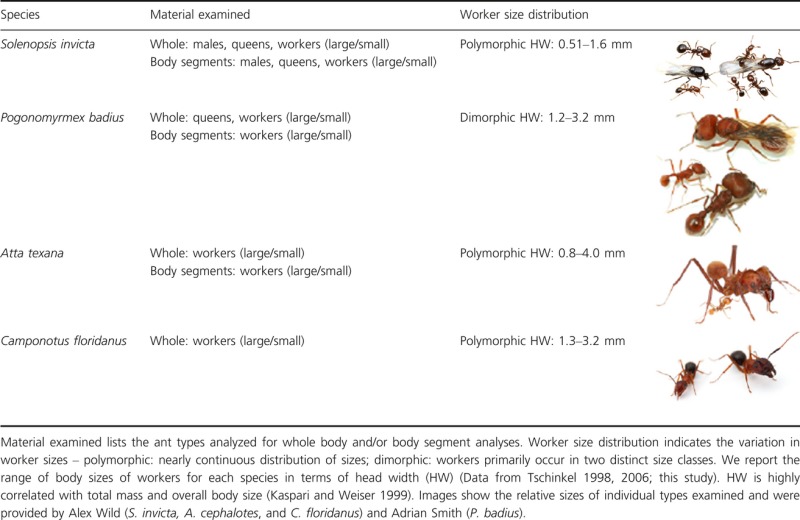

To assess the relationship between endoreduplication and worker body size, we sampled the largest and smallest workers from each of the four species. To assess the relationship between endoreduplication and caste, we used whole individuals of each female type (alate queens, small workers, and large workers) of *S. invicta* and *P. badius*. For *S. invicta*, we were also able to sample males allowing for the comparison between diploid (females) and haploid (male) individuals. Finally, to examine patterns of endoreduplication among different body parts within individuals, small and large workers of *S. invicta*, *A. texana*, and *P. badius*, and alate queens and males of *S. invicta* were divided into head (including antenna), thorax (including legs and wings, if present), and abdomen subsamples. We do note that in the order Hymenoptera, the first abdominal segment is fused to the thorax and forms the “mesosoma” (mesosoma = thorax plus abdominal segment one). For the sake of simplicity, we refer to this structure as the thorax here. Similarly, when we refer to the abdomen, it is actually the “metasoma” which includes all abdominal segments except the first one.

For whole-individual samples, multiple individuals of each type per species were pooled into single samples to obtain enough tissue per sample for analysis. Between five and 11 samples per type were analyzed for each species and the samples within each species were standardized for mass by analyzing an equivalent quantity of tissue for each sample. As with whole individuals, multiple subsamples of each body part per caste/size class for each species were pooled into single samples and the samples within species were standardized for mass (Table 1).

### Cytometric analysis

**Figure 1:**
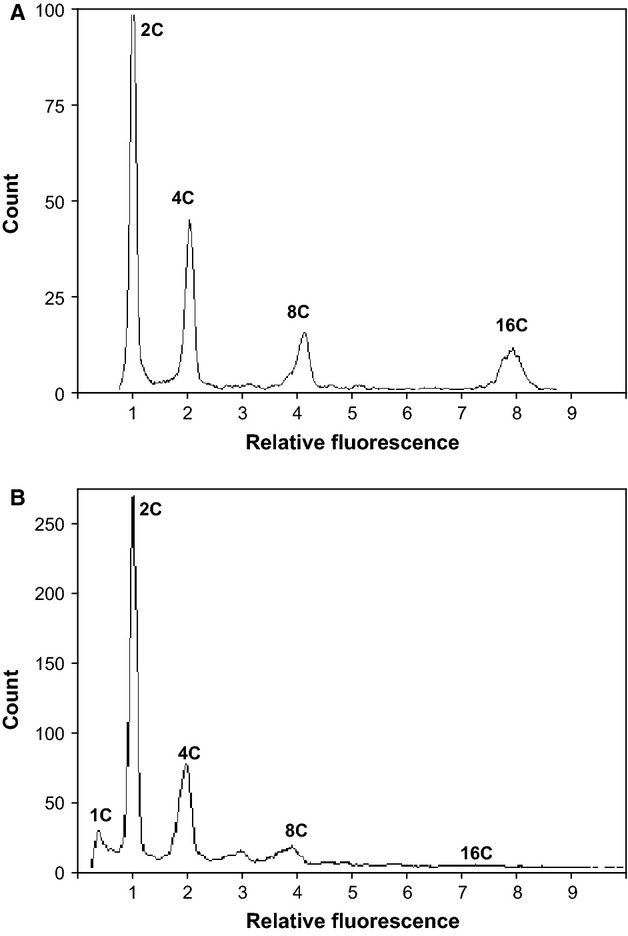
Flow cytometry histogram of (A) an *Atta texana* large worker whole-body sample and (B) a *Solenopsis invicta* male head sample. Relative fluorescence approximately doubles with each DNA replication phase. The small peaks at approximately 3 and 6 relative fluorescence are cells temporarily arrested at the intra-S phase checkpoint.

Tissue for flow cytometric analysis was prepared via standard protocols (see Galbraith et al. 1983). In brief, fresh ant tissue was chopped with a razor blade, sheared in a nuclear isolation buffer (sodium citrate, 3-morpholinopropane-1-sulfonic acid, magnesium chloride, Triton X-100; Galbraith et al. 1983), filtered for debris removal, and stained with propidium iodide. Suspended nuclei were analyzed via a BD Biosciences (Franklin Lakes, NJ) FACScanto flow cytometer for measurement of nuclear DNA content. Background correction for debris removal and nuclei population gating were performed using De Novo Software FCS Express (v.3, Los Angeles, CA) to measure the number of nuclei at each ploidy level (1C, 2C, 4C, 8C, 16C) per sample (see Fig. 1 for sample histograms). Due to the wide range of fluorescence measures (proportionally from 1°C to 16°C DNA content), a high-resolution calibration sample was used to estimate nuclei populations for samples with reduced resolution at one or both ends of the fluorescence scale. The cycle value, calculated as the mean number of endoreduplication cycles per nucleus and thus an overall measure of endoreduplication, was calculated from the number of nuclei at each ploidy level detected for each flow cytometric sample as described by Barow and Meister (2003) (Fig. 1). Cell cycle values of females were calculated by the following equation:


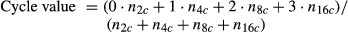


as the sum of the number of nuclei at each ploidy level detected multiplied by the number of endocycles required to achieve each corresponding ploidy level, divided by the total number of nuclei measured. Because males have haploid cells, the cell cycle value calculation of males was modified such that:


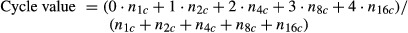


### Statistical analysis

All statistical analyses for cycle values were conducted as mixed models with SAS (v.9.2, Cary, North CA). Cycle values of whole-individual samples were analyzed with species as a random effect and individual type (male, queen, large worker, and small worker) as a fixed effect. Contrasts were designed to test for overall effects of caste and female body size, with pairwise contrasts between types of individuals within each species corrected for family-wise error rate by Bonferroni corrections when multiple comparisons were performed within a species (Rice 1989). Cycle values of body part samples were analyzed with species as a random effect and individual type and body part as fixed effects. Contrasts were designed to test for overall within- and between-type effects, with pairwise contrasts between body parts within types and between types within body parts corrected for family-wise error rate by Bonferroni corrections when multiple comparisons were performed within a type/body part (Rice 1989).

## Results

### Whole-individual model: species and type

Endoreduplication does not differ for females of different species (*S*. *invicta* Q, LW, SW vs. *P*. *badius* Q, LW, SW vs. *A*. *texana* LW, SW vs. *C. floridanus* LW, SW, *F*(3,3.98) = 3.17, *P* = 0.148), but does differ between different types of females across all species (*S*. *invicta*, *P*. *badius* Q vs. *S*. *invicta*, *P*. *badius*, *A*. *texana*, *C*. *floridanus* LW vs. *S*. *invicta*, *P*. *badius*, *A*. *texana*, *C*. *floridanus* SW, *F*(3,3.88) = 56.07, *P* < 0.01) with a significant species*type interaction (*F*(4,77) = 2.74, *P* < 0.05).

### Ploidy and worker body size: whole individuals

**Figure 2:**
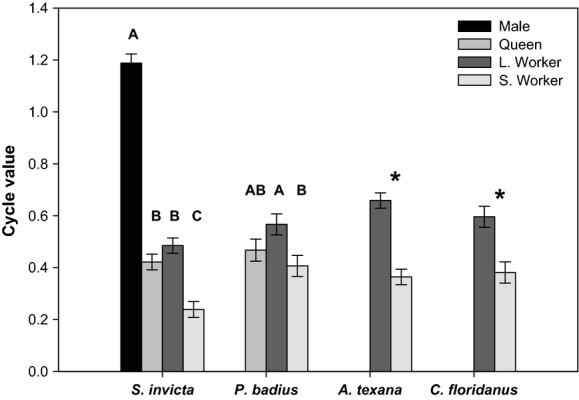
Cycle values of whole-body samples of males, queens, large workers, and small workers of each species for which they were sampled. Shown are means ± SE. Letters indicate significance among means in each comparison group and asterisks indicate significance between two means. All significance was determined at *α* = 0.05.

Across all species, endoreduplication is greater for large workers than for small workers (*S*. *invicta*, *P*. *badius*, *A*. *texana*, *C*. *floridanus* LW>SW, *F*(1,4) = 25.03, *P* < 0.01; Fig. 2). This pattern holds for each species when comparing worker sizes for each species independently (LW>SW; *S*. *invicta*, *F*(1,4) = 35.96, *P* < 0.01; *P*. *badius*, *F*(1,4) = 8.98, *P* < 0.05; *A*. *texana*, *F*(1,4) = 53.51, *P* < 0.01; *C*. *floridanus*, *F*(1,4) = 16.14, *P* < 0.05; Fig. 2).

### Ploidy and caste: whole individuals

Within *S*. *invicta*, for which males and all three types of females were sampled, endoreduplication differed between reproductive and nonreproductive castes (M, Q vs. LW, SW, *F*(1,4) = 198.35, *P* < 0.001; Fig. 2). Excluding males, however, endoreduplication does not differ between reproductive and nonreproductive females for the two species for which both reproductive and nonreproductive females were sampled (*S*. *invicta*, *P*. *badius* Q vs. LW, SW, *t*(4) = 0.61, *P* = 0.575; Fig. 2). This pattern is driven by similarities between queens and large workers; in pair-wise comparisons, endoreduplication did not differ between alate queens and large workers (*S*. *invicta*, *P*. *badius* Q = LW, *F*(1,4) = 4.58, *P* = 0.099), but did differ between alate queens and small workers (*S*. *invicta*, *P*. *badius* Q>SW, *F*(1,4) = 10.19, *P* < 0.05) and between large workers and small workers (*S*. *invicta*, *P*. *badius* LW>SW, *F*(1,4) = 32.45, *P* < 0.01; Fig. 2).

### Ploidy and body segments

Across all three species where body parts were analyzed in workers (*S*. *invicta*, *P*. *badius*, and *A*. *texana)*, endoreduplication of large and small workers differed among body parts (*F*(2,4) = 92.46, *P* < 0.001). Specifically, for large workers, endoreduplication is greatest in the abdomen, with no differences between heads and thoraces (abdomen vs. head, *t*(4) = 7.9, *P* < 0.01; abdomen vs. thorax, *t*(4) = 6.77, *P* < 0.01; head vs. thorax, *t*(4) = 1.13, *P* = 0.323). This same pattern is present for small workers (SW abdomen>head = thorax; abdomen vs. head, *t*(4) = 13.27, *P* < 0.001; abdomen vs. thorax, *t*(4) = 12.32, *P* < 0.001; head vs. thorax, *t*(4) = 0.95, *P* = 0.3978). For *S*. *invicta* queens, endoreduplication did not differ between any body parts (head vs. thorax, *t*(4) = 0.11, *P* = 0.921; head vs. abdomen, *t*(4) = 2.08, *P* = 0.1059; thorax vs. abdomen, *t*(4) = 1.98, *P* = 0.119). However, in *S*. *invicta* males, endoreduplication was lowest in the thorax with no difference in the abdomen and head (head vs. abdomen, *t*(4) = 0.09, *P* = 0.931; head vs. thorax, *t*(4) = 2.83, *P* < 0.05; abdomen vs. thorax, *t*(4) = 2.92, *P* < 0.05).

**Figure 3:**
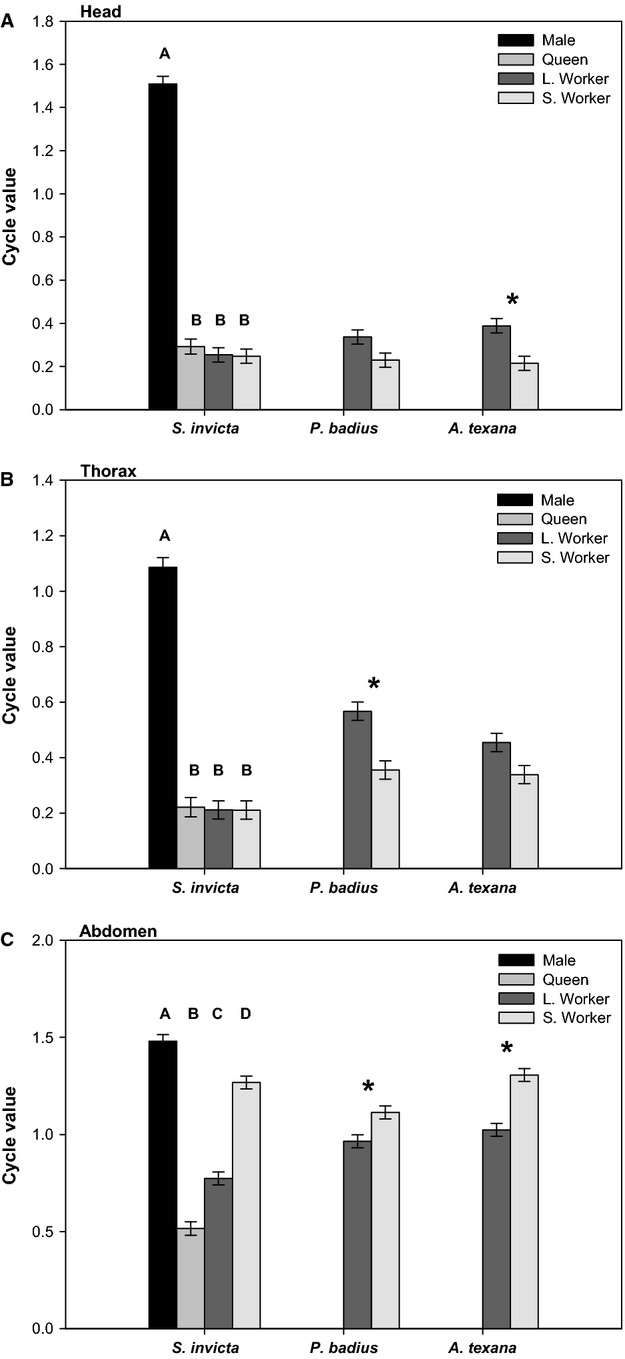
Cycle values of (A) heads, (B) thoraces, and (C) abdomens of males, queens, large workers, and small workers of each species for which they were sampled. Shown are means ± SE. Letters indicate significance among means in each comparison group and asterisks indicate significance between two means. All significance was determined at *α* = 0.05.

In the body part model, there was also a significant species*type*part interaction (*F*(4,84) = 6.26, *P* < 0.001) for female samples. This appears primarily driven by differences between *S*. *invicta* and the other two species sampled (*P. badius* and *A. texana)*. Specifically, while there were no significant differences in endoreduplication between *S*. *invicta* queen, large worker, and small worker heads and thoraces (Q head = LW head = SW head; Q vs. LW *F*(1,4) = 0.65, *P* = 0.4665; Q vs. SW, *F*(1,4) = 0.87, *P* = 0.4037; LW vs. SW, *F*(1,4) = 0.02, *P* = 0.899; (Fig. 3A)) (Q thorax = LW thorax = SW thorax; Q vs. LW, *F*(1,4) = 0.04, *P* = 0.852; Q vs. SW, *F*(1,4) = 0.05, *P* = 0.8372; LW vs. SW, *F*(1,4) = 0.001, *P* = 0.984 (Fig. 3B)), endoreduplication of the *A*. *texana* head and the *P*. *badius* thorax of large workers is greater than those of small workers (*A. texana* LW head>SW head: *F*(1,4) = 14.5, *P* < 0.05 (Fig. 3A)) (*P. badius* LW thorax>SW thorax: *F*(1,4) = 21.44, *P* < 0.01; (Fig. 3B)). Furthermore, for *S*. *invicta* in which all types of females were sampled, endoreduplication is greatest in the small worker abdomen, followed by the large worker abdomen, and the lowest in the queen abdomen (SW abdomen>LW>Q; SW vs. LW: *F*(1,4) = 117.15, *P* < 0.001; LW vs. Q: *F*(1,4) = 28.97, *P* < 0.01; SW vs. Q: *F*(1,4) = 246.78, *P* < 0.001; Fig. 3C). The same trend is evident between worker abdomens of *P. badius* and *A. texana* (SW abdomen>LW; *P. badius*: *F*(1,4) = 10.55, *P* < 0.05; *A. texana*: *F*(1,4) = 38.37, *P* < 0.01; Fig. 3C). Endoreduplication was greater in *S*. *invicta* males than for any other individual, regardless of tissue (Fig. 3).

## Discussion

By examining patterns of within-individual ploidy in four species of ants, we found that endoreduplication is positively related with size within the worker caste, but not related to caste generally in those species for which reproductives were sampled. Specifically, queens of *S. invicta* and *P. badius* exhibited patterns of endoreduplication similar to the largest workers. When separating individuals into their three primary body segments, we found that abdomens had the greatest endoreduplication of all body parts in females regardless of individual type (caste or size; though not significant for *S*. *invicta* queens). We also found some differences among species in patterns of endoreduplication across body parts. Additionally, we found that males exhibited greater endoreduplication than all types of females in *S. invicta*.

Greater whole-individual endoreduplication was observed in large workers compared to small workers for each species examined. This pattern indicates that endoreduplication and its associated nucleotypic and/or genetic effects may be a generally conserved characteristic promoting larger size within the worker caste. We did not find an effect of caste (queen vs. worker) on endoreduplication. This may be due to the promotion of overall body size by endoreduplication or possibly by the endoreduplication of type-dependent tissues that create an overall signature of high levels of endopolyploidy. In *S*. *invicta,* variation in worker mass has been linked to the expression of at least one gene (Goodisman et al. 1999). Future work linking endoreduplication with gene-specific expression patterns is needed to determine if endopolyploidy is a common mechanism for promoting increased size in ants.

In addition to differences in overall size, workers of difference size classes often exhibit different behaviors (Wilson 1953, 1980; Robinson 1992; Beshers and Traniello 1996; Tschinkel 2006; Mertl and Traniello 2009). Therefore, differences in endoreduplication may be correlated with behavior and promote division of labor. For example, the largest workers may be particularly suited for nest defense and specialized foraging roles (Hölldobler and Wilson 1990; Wilson 2003; Powell and Franks 2005; Powell 2008), whereas small workers may be more suited for nest maintenance, tending to the queen and brood care (Wilson 1980; Hölldobler and Wilson 1990; Tschinkel 2006). Based on the relationship between endoreduplication and size, worker specialization could be driven in part by endoreduplication-induced differences in gene expression (Nagl 1976; Barow 2006). If endopolyploidy is an efficient means to meet the proteomic demands of specialized cells by stimulating targeted increases in gene expression (Larkins et al. 2000), future examination of the role of ploidy in age, size, and caste-specific patterns of gene expression is warranted (e.g., Whitfield et al. 2003; Toth et al. 2007; Graff et al. 2007; Ometto et al. 2011).

Of all major body segments, the abdomen showed the greatest levels of endopolyploidy regardless of species or caste (except for *S*. *invicta* males; see Fig. 3), indicating that endoreduplication is an important process in the maturation and function of the various cell types within. Our results showing the abdomen has high levels of endopolyploidy relative to other parts supports the hypothesis that endopolyploidy may be common in metabolically costly tissues such as those found in the highly specialized and internally complex abdomen (Aron et al. 2005). For example, in *Drosophila melanogaster*, the Malpighian tubules, which are specialized to absorb water, solutes, and wastes and excrete them as nitrogenous compounds, are known to endoreduplicate up to 256°C (Lamb 1982). Even mammals, which are almost universally diploid and have relatively little endoreduplication in their tissues, undergo endoreduplication as an important process in the terminal differentiation and gene regulation of the liver hepatocyte cells (Brodsky and Uryvaeva 1977; Anatskaya and Vinogradov 2010). These studies collectively suggest that the metabolic demands of digestion, excretion, and other specialized functions may be met by endoreduplication, and may also explain the high levels of endopolyploidy observed in the abdomen.

While reproductive individuals may be expected to have the greatest abdominal endoreduplication due to their large and highly metabolic reproductive tissues, our results indicate that reproductive organs are not the only sources of endoreduplication in ant abdomens. Because the abdomen contains the digestive and excretory organs, and numerous hypertrophied glands related to chemical communication and venom production, endoreduplication may be particularly important for the functional maturation of these specialized tissues in workers. Small workers in particular are the primary scouts of the ant colony, and therefore frequently communicate with nest mates and define foraging trails via pheromone production of the ventral venom, poison, sternal, pygidial, Dufour's, and other abdominal glands (Chapman 1998). The high degree of metabolism and specialization of these worker abdominal tissues may be supported by their high levels of endopolyploidy, whereas the reproductive tissues of the *S*. *invicta* queen abdomen sampled here may not be as highly metabolic as first assumed; recall that the queens sampled here were alate, unmated queens and therefore may not have been active in egg production at the time of sampling. The patterns observed here where abdominal endopolyploidy is greatest in small workers, followed by large workers, and queens with the least may perhaps be the result of the combination of worker societal role and queen developmental stage described above. We also caution that the approximate reconstruction of the whole-body results via the body part analysis is not expected as the whole-body mass is not evenly distributed across the three body parts. Therefore, the body part results must be considered with respect to the relative sizes and internal composition of each part within and across individual types to understand how the endopolyploidy of each part contributes to the whole-body values observed.

Males of *S. invicta* had greater endoreduplication in both whole body and body part samples than any female sampled. The difference in endoreduplication between the sexes may be related to the haplodiploid sex determination system of Hymenoptera, where females develop from fertilized eggs and males from unfertilized eggs. Endoreduplication has been hypothesized to overcome negative metabolic and gene dosage effects due to low gene copy number (Epstein 1964, 1967), which would be likely disadvantages of being haploid. The fact that males endoreduplicate at such high rates, nearly averaging an additional full endoreduplication cycle compared to females (cycle value for males: 1.2563, females: 0.4657) supports this hypothesis. Aron et al. (2005) reported that nuclear DNA is similarly restored in males of all hymenopteran lineages examined except the most basal one (Xyelidae), suggesting that the adaptation of increased endoreduplication in males arose early in the evolution of Hymenoptera and is generalizable to the order. It should also be noted that introduced populations of *S. invicta* often produce diploid males (Ross and Fletcher 1985) that can be sterile or lead to the production of triploid females (Krieger et al. 1999). However, our cytometric analyses of males suggest this is not the case in our study population as many cells were still haploid (Fig. 1B). Additional work comparing rates of endopolyploidy between males and females across many species of ants is needed and will be valuable particularly if individual tissue types are compared (e.g., Aron et al. 2005).

While cycle values of the abdomen were substantially higher than the head and thorax for all female workers sampled (recall no significant difference for *S*. *invicta* queens), the mean cycle value of the male head was comparable to that of the abdomen. This result suggests that endoreduplication is particularly important in the specialization of some tissues therein; the male head is small in size relative to its other parts and the ratios observed in females and so the relatively high levels of endoreduplication observed within are not likely functioning to generate size. Zube and Rössler (2008) observed that whereas *C*. *floridanus* females all lacked serotonergic innervations in their antennal lobes and had the same number of glomeruli, males had extensive innervations that support their heightened olfactory perception. Olfactory, as well as visual perception, is particularly important for males as they establish nuptial sites and points to specific tissues that may be driving this pattern in males but remain relatively undeveloped in females.

Because endoreduplication occurs widely throughout Eucarya, the role of endoreduplication has been hypothesized to be quite general (Nagl 1976). The long-standing hypothesis is that endoreduplication is involved in organismal growth via increases in cell size and gene copy number, and thus provides an energetically efficient means of increasing organism and/or tissue size and metabolism without the expense of mitosis (Kondorosi et al. 2000). Our results in ants, where size and caste are predominately determined by nutrition (Wheeler 1986, 1991), are consistent with this hypothesis.

The patterns of endopolyploidy we report provide evidence for a link between endoreduplication and worker body size in ants, and perhaps to energetics (namely the high levels observed in all abdomens) as well. To more fully substantiate these links, intrasegment tissue sampling could help explain the patterns observed, and additional species in other taxa should also be examined. For example, endoreduplication has been associated with body size in the noneusocial, endoparasitic insect order Strepsiptera (Johnston et al. 2004). Moreover, analyses of individual organs or organ systems, particularly of the abdomen, are needed to determine which tissues are driving the patterns of endopolyploidy we report for whole individuals and for specific body parts. The next step for this research will be to combine measures of endopolyploidy with examinations of gene expression both among species and specific tissues. With at least seven ant genomes now published, including four polymorphic species (Gadau et al. 2012), this should prove to be a promising avenue for future research. While challenging, uncoupling the effects of endoreduplication on size, caste, and behavior in social insects in the order Hymenoptera may provide insight into the molecular mechanisms of size-based division of labor and how haploid sexes are able to meet the proteomic demands of their cells.
